# Safety and Outcomes of Peripherally Administered Vasopressor Infusion in Patients Admitted with Shock to an Intensive Cardiac Care Unit—A Single-Center Prospective Study

**DOI:** 10.3390/jcm12175734

**Published:** 2023-09-03

**Authors:** Elad Asher, Hani Karameh, Hamed Nassar, Chaim Yosefy, David Marmor, Nimrod Perel, Louay Taha, Meir Tabi, Omri Braver, Mony Shuvy, Yonit Wiener-Well, Michael Glikson, Sharon Bruoha

**Affiliations:** 1 Jesselson Integrated Heart Center, Shaare Zedek Medical Center and Faculty of Medicine, Hebrew University of Jerusalem, Jerusalem 91121, Israel; easher@szmc.org.il (E.A.); hanik@szmc.org.il (H.K.); hnccu@szmc.org.il (H.N.); davidma@szmc.org.il (D.M.); n_perel@yahoo.co.in (N.P.); louayt@szmc.org.il (L.T.); meirta@szmc.org.il (M.T.); monysh@szmc.org.il (M.S.); mglikson@szmc.org.il (M.G.); 2 Department of Cardiology, Barzilai Medical Center, The Ben-Gurion University of the Negev, Beer-Sheva 84105, Israel; yosefy@bmc.gov.il (C.Y.); omribr@bmc.gov.il (O.B.); 3 Infectious Diseases Unit, Shaare Zedek Medical Center and Faculty of Medicine, Hebrew University of Jerusalem, Jerusalem 91121, Israel; yonitw@szmc.org.il

**Keywords:** cardiogenic shock, vasopressors, central venous catheter, peripheral venous catheter

## Abstract

Background: Vasopressors are frequently utilized for blood pressure stabilization in patients with cardiogenic shock (CS), although with a questionable benefit. Obtaining central venous access is time consuming and may be associated with serious complications. Hence, we thought to evaluate whether the administration of vasopressors through a peripheral venous catheter (PVC) is a safe and effective alternative for the management of patients with CS presenting to the intensive cardiovascular care unit (ICCU). Methods: **A** prospective single-center study was conducted to compare the safety and outcomes of vasopressors administered via a PVC vs. a central venous catheter (CVC) in patients presenting with CS over a 12-month period. Results: **A** total of 1100 patients were included; of them, 139 (12.6%) required a vasopressor treatment due to shock, with 108 (78%) treated via a PVC and 31 (22%) treated via a CVC according to the discretion of the treating physician. The duration of the vasopressor administration was shorter in the PVC group compared with the CVC group (2.5 days vs. 4.2 days, respectively, *p* < 0.05). Phlebitis and the extravasation of vasopressors occurred at similar rates in the PVC and CVC groups (5.7% vs. 3.3%, respectively, *p* = 0.33; 0.9% vs. 3.3%, respectively, *p* = 0.17). Nevertheless, the bleeding rate was higher in the CVC group compared with the PVC group (3% vs. 0%, *p* = 0.03). Conclusions: **The administration** of vasopressor infusions via PVC for the management of patients with CS is feasible and safe in patients with cardiogenic shock. Further studies are needed to establish this method of treatment.

## 1. Introduction

Intravenous (IV) vasopressors are frequently utilized in critically ill patients with circulatory shock to raise their blood pressure and improve organ perfusion [1]. Vasopressors are commonly given through a central venous catheter (CVC) due to historical concerns for complications associated with the vasoconstrictive properties when administered via a peripheral venous catheter (PVC), such as skin ischemia and necrosis [2]. Accordingly, the administration of vasopressors via PVCs has been traditionally discouraged, whereas the use of CVC access has been repeatedly advocated. However, CVC insertion is not free of risks, with a reported complication rate as high as 20% [3]. Complications can present in an immediate or delayed fashion and vary based on the type of central venous access. For instance, the subclavian venous route is associated with the highest risk for pneumothoraces and hemothoraces and the lowest rate of infections compared to the femoral and internal jugular venous routes [4].

An inadvertent arterial puncture or cannulation, lung injuries, local and systemic infections, and venous thrombosis are among the most commonly associated adverse events [5]. Overall, the number of unsuccessful insertion attempts is the biggest predictor of complications. Ultrasound guidance has been shown to decrease the risk of complications at all access sites, especially vascular complications [6].

Furthermore, CVC insertion is time consuming and may hinder the prompt initiation of vasopressor therapy [7]. Delays in vasopressor administration in the setting of shock have even been associated with increased mortality [8]. However, establishing a dedicated central venous line service is associated with a shorter time to central venous access and the initiation of the vasopressor [9]. Both noradrenaline and dopamine are recommended as first-line vasoactive agents for shock [10]. However, noradrenaline appears to be safer for CS. In a study that compared dopamine and noradrenaline for the treatment of shock in 1679 patients, of which 280 patients had CS, dopamine was associated with more arrhythmic events, especially atrial fibrillation, than noradrenaline. However, in a subgroup analysis according to the type of shock, dopamine was associated with a higher mortality in patients with shock due to cardiac failure [10]. Norepinephrine is a potent sympathomimetic agent acting on α and β receptors. The alpha-adrenergic effect increases the vascular tone, but may decrease the regional blood flow to the skin and viscera [11].

Contemporary observational studies and meta-analyses have reported conflicting data regarding the occurrence of local adverse events due to peripheral vasopressor extravasation. Several studies have demonstrated a relatively low incidence of complications and morbidity associated with vasopressors infused via PVCs [2,12,13,14,15], though most of them were observational or retrospective studies and included patients presenting with different kinds of shock or post-surgery. For instance, in a meta-analysis that included 1835 patients with shock that were treated with vasopressors via PVCs, 122 (7%) complications occurred, of which 117 (96%) were minor [2]. In contrast, another study that included 734 unstable patients reported local adverse events in 19 (2%) patients due to the peripheral administration of vasoactive medications. Hence, we sought to prospectively evaluate the safety and efficacy of vasopressors introduced via PVCs in patients presenting mainly with CS who were admitted to a contemporary tertiary care medical center ICCU.

## 2. Methods

### 2.1. Study Population

All patients were admitted to a tertiary care medical center ICCU at the Shaare Zedek Medical Center (Jerusalem, Israel) over a 12-month period between January 2022 and December 2022. Vasopressors for the treatment of shock were administered either via a CVC or via a PVC. The decision to administer vasopressors via a CVC or via a PVC was made according to the discretion of the treating senior cardiologist. Of note, the administration of vasopressors through a peripheral line is the standard approach in our center for the initial management of patients suffering from shock. However, when additional medications with potential toxicity, multiple vasopressors, fluids at a high rate, and/or blood products are co-administered, a CVC is generally preferred. Hence, overall, patients with a central venous access are in a higher degree of shock compared to patients with a peripheral vascular access. A vasopressor treatment was administered in accordance with the European Society of Cardiology (ESC) guidelines for heart failure in patients with cardiogenic shock [16] and with the Surviving Sepsis Campaign (SSC) international guidelines for the management of sepsis and septic shock [17]. Noradrenaline was the vasopressor of choice.

Inclusion criteria: All patients were ≥18 years old and presented with hemodynamic shock requiring vasopressor administration.

Exclusion criteria: Patients <18 years old and pregnant women were excluded from the study.

Definitions:

1. Cardiogenic shock was defined as a systolic blood pressure (SBP) <90 mm Hg that was refractory to fluid resuscitation with clinical and laboratory evidence of end-organ dysfunction in the setting of suspected cardiac dysfunction and/or right heart catheterization with a cardiac index (CI) of ≤2.2 L/min per m^2^ and a pulmonary capillary wedge pressure (PCWP) of ≥15 mm Hg [18].

Patients with CS were further classified according to the Society for Cardiovascular Angiography and Interventions (SCAI) shock classification to A, B, C, D, or E [19].

2. Septic shock was defined as a suspected or confirmed infection, plus hyperlactatemia (≥2.0 mmol/L), and the requirement of vasopressors to maintain a mean arterial pressure (MAP) of 65 mm Hg or higher after an IV fluid load of at least 20 mL/kg over 60 min [20].

3. Sepsis-induced decompensation together with an acute myocardial infarction or chronic heart failure was defined as combined shock.

Vasopressor administration: vasopressors were administered under strict invasive blood pressure monitoring for the shortest period necessary. The PVC was placed preferably in a vein proximal to the wrist via (at least) a 20 G cannula. A second peripheral venous access was routinely obtained for backup and the administration of other IV drugs. The CVC was inserted under ultrasound guidance and after a strict sterile preparation technique. Each vasopressor was given according to specific protocols that included the drug dilution, concentration, and rate of initial and maximal pressor dose. An inspection of the venous access site was performed daily.

### 2.2. Data Collection

Data were prospectively and anonymously documented in an electronic case report form (eCRF). The data were checked for accuracy and out-of-range values by the study coordinator.

The demographic data, presenting symptoms, comorbid conditions, and a physical examination were systematically recorded. Laboratory, imaging, hemodynamic, and clinical course data were collected as well. Patients with shock were stratified according to the vasopressor administration route (peripheral vein vs. central vein), the decision of which was made according to the discretion of the treating senior cardiologist.

The vasopressor type, the concentration, and the duration of administration were all recorded as well.

The study was approved by the SZMC Institutional Review Board (approval number 0431-21-SZMC; approved on 28 February 2022) with an exemption from informed consent.

### 2.3. Study Outcomes

The primary outcomes included: a central-line-associated blood stream infection (CLABSI) (presented as a CLABSI divided by central line days), a blood stream infection (BSI) (presented as a BSI divided by peripheral line days), phlebitis, extravasation, and bleeding.

The secondary outcomes included: the hospitalization length and the mortality rate. The mortality rate was derived from The Israeli Ministry of Internal Affairs medical record database, which is continuously updated by every medical center in Israel.

### 2.4. Statistical Methods

The patients’ characteristics were presented as numbers (%) for categorical variables, and as means (SD) or medians (IQR) for normally and non-normally distributed continuous variables, respectively. A comparison of categorical variables was achieved using a chi-squared test and Fisher’s exact test. Student’s *t*-test and the Mann–Whitney test were performed for the comparison of normally and non-normally distributed continuous variables, respectively.

Mortality was analyzed by applying a stepwise backward Cox proportional hazards model. All tests were two-sided. *p* < 0.05 was considered statistically significant. The analyses were carried out using SPSS Statistics for Windows, version 25.0 (IBM Corp, Armonk, NY, USA).

## 3. Results

### 3.1. Patient Characteristics

A total of 1100 patients were included in the study. Of them, 139 (12.6%) required vasopressor treatment due to shock. Patients in the CVC group were, on average, 8 years younger than those in the PVC group (72 (±12.3) years old vs. 64 (±19.6) years old, respectively, *p* < 0.01), while the proportion of people belonging to the female gender was similar in both groups. Patients in both groups had similar rates of diabetes mellitus (DM), hypertension, dyslipidemia, prior coronary artery disease, peripheral artery disease, and cerebrovascular accidents. The etiologies for shock were: cardiogenic, 120 (86%); septic, 11 (8%); combined (cardiogenic and septic), 5 (4%); and hemorrhagic, 3 (2%). The patient characteristics and shock types are presented in Table 1. Patients in the CVC group suffered from higher stages of shock compared to those in the PVC group.

### 3.2. Characteristics of Venous Access and Vasopressor Treatment

The drug therapy was administrated via a PVC in 108 (78%) patients and via a CVC in 31 (22%) patients. A peripheral IV cannula was placed above the wrist in all patients. A CVC was placed in the jugular vein in nearly half (45%) of the cases, followed by the femoral vein (29%) and subclavian vein (26%). One hundred and eleven patients (80%) were successfully managed with one vasopressor. The most administered vasopressor agent was noradrenaline (94%), followed by dopamine (12%). The characteristics of the venous access and vasopressor treatment are presented in Table 2 and Table 3, respectively. The characteristics of the noradrenaline treatment are presented in Table 4.

### 3.3. Complications during Admission

The adverse events during hospitalization are reported in Table 5. The overall local complication rate was 9% of patients and was similar in both groups (four (13%) events in the CVC group and nine (8%) in the PVC group, *p* = 0.8). Phlebitis and extravasation occurred at similar rates in both groups (3% vs. 5% in the CVC and PVC groups, respectively, and 3% vs. 2% in the CVC and PVC groups, respectively). Although the total number of events was small, there was a higher rate of local adverse events seen at higher doses (41–60 mcg/min) of noradrenaline when compared to lower doses. All cases were managed conservatively.

A CLABSI occurred in only one patient with a CVC (3.3/1000 central catheter days) and a BSI occurred in only two patients with a PVC (2.8/1000 peripheral catheter days), with *p* = 0.5.

### 3.4. Length of Vasopressor Treatment and Admission

Overall, 406 days of vasopressor treatment were monitored during the study period. The duration of the vasopressor treatment was shorter in the PVC group compared to the CVC group (2.5 days vs. 4.2 days, respectively, *p* < 0.05). The mean length of admission was shorter in the PVC group (8.3 days vs. 5.67 days, respectively, *p* = 0.001).

### 3.5. Mortality Rate

The in-hospital mortality rate was 20% (28/139), with a higher mortality rate in the CVC group compared to the PVC group (36% (11/31) vs. 16% (17/108), respectively, *p* = 0.007).

## 4. Discussion

The present study sought to evaluate the safety and efficacy of vasopressors introduced via a PVC in patients presenting mainly with CS to a tertiary care medical center ICCU. To our knowledge, this is the first study to show the safety and feasibility of vasopressors administered at conventional doses via a PVC in patients with CS managed in a contemporary ICCU. Our main findings were the following: (1) the administration of vasopressors via a PVC is feasible in patients with less severe degrees of shock and (2) the administration of vasopressors via a PVC is safe, with a lower incidence of local adverse events when compared to a CVC.

CS is the leading cause of death in acute myocardial infarctions (AMIs), and it is characterized by tissue hypoperfusion and hypoxia related to a low cardiac output [21]. It is often associated with rapid hemodynamic deterioration, unresponsiveness to intensive supportive measures, and a high mortality rate [22].

Nationwide databases examining the temporal trends in CS have shown inconsistent data regarding the incidence of CS. While some studies demonstrate an increase in the overall incidence of CS in recent years [23], others report a decrease [24,25].

CS complicates approximately 5–10% of AMIs, with a higher incidence in ST-elevation myocardial infarctions, and it is more frequently seen among women and patients >75 years old [18,23].

The clinical and hemodynamic heterogenicity of CS, with only a few randomized clinical trials evaluating the various therapeutic approaches and recommendations, leads to uncertainties as to the best treatment strategies. Thus, the management of CS is often challenging and requires an early diagnosis and the institution of high-quality interdisciplinary care [26]. When treated conservatively, CS carries a ~70–80% risk of mortality [27]. In contrast, early reperfusion has been associated with improvements in survival [28]. However, for more than two decades, the in-hospital and 1-year mortality rates remained unchanged and unacceptably high, with a reported rate of 40–50% [29]. The current management recommendations for CS are based on early revascularization along with general supportive measures, such as fluids and oxygenation, vasopressors and inotropes, and the use of temporary mechanical support (MCS) devices [16]. Early revascularization is strongly advised in CS and represents the most important intervention in the treatment of cardiogenic shock in the setting of AMIs. In the SHOCK trial [28], the overall survival at the 6- and 12-month follow-ups was significantly better with early revascularization (50% vs. 37%, *p* = 0.027, and 47% vs. 34%, *p* = 0.025, respectively). Thus, in the current era, the only intervention with a proven mortality benefit in CS complicating AMIs is early revascularization, either with a percutaneous coronary intervention or a coronary artery bypass grafting surgery with a class I indication in contemporary guidelines [16]. MCS is a relatively new option for treating CS and may offer significant advantages over drug therapy, including targeted cardiovascular support without an increased risk of myocardial ischemia, a possible reduction in the myocardial oxygen demand, and the avoidance of systemic adverse events [30]. Short-term percutaneous support platforms are widely used in the setting of CS, particularly for patients refractory to medical therapy, either alone or in combination. Most MCS insertions require large-bore vascular access [31].

Vasopressors are an essential part of the treatment for patients with shock and should be initiated early in septic shock [32]. In contrast, the benefits of vasopressor drugs for the management of CS are less established [16]. In the European heart failure guidelines, inotropes have a class IIb level of recommendation and may be considered in CS when a low systolic blood pressure (<90 mmHg) is coupled with signs of hypoperfusion [16]. Moreover, their potential adverse effects (e.g., arrhythmias and systemic vasoconstriction) and the lack of consistent evidence of a benefit mandates their cautious administration [33].

Nevertheless, vasopressors are frequently administered in patients with CS and hypotension to maintain vital organ perfusion [16]. Thus, reliable venous access is of paramount importance to ensure effective and safe drug administration, although there are no available data to support a specific route for drug delivery. Nevertheless, the local adverse event rate in our analysis was low in both groups, and all adverse events were treated conservatively.

Similar to our analysis, studies evaluating the administration of vasopressor drugs in critically ill patients via peripheral venous access reported a low overall risk for local complications (4–7%) without significant morbidity [15,34,35].

However, in contrast to previous studies that included heterogeneous groups of patients, our study included almost exclusively (86%) patients in CS. Shock secondary to a low cardiac output is a complex clinical entity associated with unique challenges. For instance, MCS devices introduced via large-bore vascular access are emerging as a cornerstone in the management of CS [31], though access complications are a significant potential drawback. The feasible delivery of vasopressors via a PVC would obviate the need for central venous access, leaving intact potential access sites for use if needed. Furthermore, CS may be associated with systemic compensatory vasoconstriction secondary to a low cardiac output [26]. Noradrenaline, a potent vasoconstrictor, infused during states of high vascular resistance may potentially induce an exaggerated increase in vascular constriction, leading to peripheral ischemia [10,36,37], especially at high doses [33,38]. In our analysis, the delivery of noradrenaline at conventional doses via a PVC was not associated with an increased risk of side effects. In contrast, at very high doses (41–60 mcg/min), noradrenaline was indeed associated with an increase in complications, although the total number of events was very small, with only four cases, and no cases of peripheral ischemia were noted. The 30-day and 1-year mortality rates for CS are approximately 40% and 50%, respectively [39]. The lower number of fatality cases seen in our analysis, especially in the PVC group, may be related to the lower CS stages compared to the CVC group. Nevertheless, even in the PVC group, more than 90% were categorized as CS with stage C or above.

Finally, the vasopressor infusion and hospitalization length were longer and the mortality rates were higher in the CVC group. Not surprisingly, we adopted a more liberal approach for CVC insertion in more complex patients. Such patients required a more aggressive treatment, including higher vasopressor doses, the administration of more than one vasopressor, fluid resuscitation, blood products, and additional IV medications. Therefore, the higher fatality rate shown in the CVC group is probably related to a selection bias.

### 4.1. Study Limitations

Our study has several limitations. First, the study was not a randomized study, and the administration of vasopressors was at the discretion of the senior cardiologist, which may have caused a selection bias, though patients in the CVC group were younger and had similar co-morbidities to the PVC group. Indeed, the longer vasopressor administration and hospitalization length and the increased mortality rate reported in the CVC group indicate a higher degree of morbidity among patients in the CVC group. Second, our primary goal was not to prove the superiority of a PVC over a CVC, but to evaluate the safety and efficacy of the PVC approach. Third, the etiologies leading to the CS were not analyzed. Finally, there was no long-term follow-up.

### 4.2. Summary and Clinical Implications

CS is a complex clinical entity associated with a high mortality rate and limited therapeutic approaches with a proven clinical benefit. Thus, the management of such patients is often challenging, and doubts as to the best treatment strategies often arise. Vasopressors are frequently infused to patients in CS, although the preferred route of administration has not been determined. A central venous line is an effective form of vascular access for the delivery of medications and fluids. However, CVC insertion requires proper training, is time consuming, and is associated with a high rate of adverse events. In contrast, the insertion of peripheral venous catheters is relatively simple with a low incidence of adverse events. Studies that have evaluated the safety and feasibility of peripherally administered vasopressors in patients with shock report a complication rate of up to 7%, with most of them minor and managed conservatively. However, prior studies included patients with different types of shock. In our analysis, the vast majority of patients had CS. In this unique population, we found that the administration of vasopressors via a PVC in CS patients is feasible and safe, at least in the initial stages of CS, with a similar, if not lower, rate of complications. Further studies are needed to establish this method of treatment. The adoption of dedicated protocols and local guidelines focusing on the management of intravascular catheters may further reduce the incidence of adverse events in patients presenting with CS.

## Figures and Tables

**Table 1:** Patient characteristics ^a^.

Clinical Variables	All Patients 139	PVC 108 (78)	CVC 31 (22)	*p*-Value
Age in years (mean ± SD)	71.6 ± 13.7	72 ± 12.3	64 ± 19.6	<0.01
Female sex—No. (%)	48 (35)	38 (35)	10 (32)	NS
BMI mean (SD)	27	27	27	NS
Hypertension	90 (65)	73 (68)	17 (54)	NS
DM	63 (45)	46 (43)	17 (54)	NS
Hyperlipidemia	71 (51)	55 (51)	16 (52)	NS
Smoking	23 (17)	16 (15)	7 (23)	NS
Prior CAD	58 (42)	45 (42)	12 (38)	NS
Prior CABG	13 (9)	10 (9)	3 (10)	NS
CVA	15 (11)	10 (9)	5 (16)	NS
PAD	9 (6)	7 (6)	2 (5)	NS
CHF or CMP	47 (34)	36 (33)	11 (35)	NS
COPD	14 (10)	9 (8)	5 (16)	NS
Atrial fibrillation	37 (27)	27 (25)	10 (32)	NS
Anemia	12 (8.5)	10 (9)	2 (5)	NS
CKD	31 (22)	27 (25)	4 (13)	NS
Shock type				
Cardiogenic shock	120 (86)	91 (84)	29 (90)	NS
Septic shock	11 (8)	11 (10)	0 (0)	
Combined	5 (4)	3 (3)	2 (6)	
Hemorrhagic	3 (2)	3 (3)	0 (0)	

PVC: peripheral venous catheter; CVC: central venous catheter; BMI = body mass index; DM = diabetes mellitus; CAD = coronary artery disease; CABG = coronary artery bypass graft surgery; CVA = cerebrovascular accident; PAD = peripheral artery disease; CHF = congestive heart failure; CMP = cardiomyopathy; COPD = chronic obstructive pulmonary disease; CKD = chronic kidney disease: NS not satisfactory. ^a^ Values reported as number (%).

**Table 2:** Characteristics of venous access ^a^.

Characteristic	Patients (139)
Peripheral venous access	108 (78)
PVC location	
Above wrist	108 (100)
Gauge	
20	108 (100)
18	0 (0)
Central venous access	31 (22)
CVC location	
Jugular	14 (45)
Femoral	9 (29)
Subclavian	8 (26)

PVC = peripheral venous line; CVC = central venous catheter. ^a^ Values reported as number (%).

**Table 3:** Characteristics of vasopressor treatment ^a^.

	All Patients 139 (100)	PVC 108 (78)	CVC 31 (22)	*p*-Value
Number of vasopressors used				*p* < 0.01
1	110 (79)	95 (88)	15 (48)	
2	22 (16)	9 (8)	13 (42)	
>2	7 (5)	4 (4)	3 (10)	
Type of vasopressor				*p* < 0.01
Noradrenaline	130 (94)	103 (95)	27 (87)	
Dopamine	16 (12)	10 (9)	6 (19)	
Phenylephrine	13 (9)	7 (6)	6 (19)	
Vasopressin	13 (9)	5 (5)	8 (26)	
Adrenaline	4 (3)	2 (2)	2 (6)	
Days of treatment				*p* < 0.001
1	34 (24)	32 (30)	2 (6)	
2	41 (29)	32 (30)	9 (29)	
3	26 (19)	21 (19)	5 (16)	
>3	38 (27)	23 (21)	15 (48)	
Days in ICCU				*p* < 0.001
1	13 (9)	12 (11)	1 (3)	
2	13 (9)	8 (7)	5 (16)	
3	12 (9)	9 (8)	3 (10)	
>3	101 (73)	79 (73)	22 (71)	

PVC = peripheral venous line; CVC = central venous catheter; ICCU = intensive coronary care unit. ^a^ Values reported as number (%).

**Table 4:** Noradrenaline treatment characteristics ^a^.

Patients Treated with Noradrenalin: 130 (94)										
	Noradrenaline via PVC: 103 (79)					Noradrenaline via CVC: 27 (21)				
Dose (mcg/min)	1–10	11–20	21–30	31–40	41–60	1–10	11–20	21–30	31–40	41–60
Patients	48 (47)	14 (14)	14 (14)	5 (5)	22 (21)	5 (19)	2 (7)	4 (15)	3 (11)	13 (48)
Duration, mean (days)	1.83	2.7	4.5	2.8	2.9	2.2	6	3.75	3.6	4.7

PVC = peripheral venous line; CVC = central venous catheter. ^a^ Values reported as number (%).

**Table 5:** Local complications associated with vasopressors ^a^.

	Patients			
Complications	Total (139)	PVC (113)	CVC (31)	*p*-Value
Phlebitis	7 (5)	6 (5)	1 (3)	NS
Extravasation	2 (1)	1 (1)	1 (3)	NS
Skin necrosis	0	0	0	NS
Line sepsis (number/1000 catheter days)	3	2 (2.8)	1 (3.3)	NS
Bleeding	1 (0.7)	0	1 (3)	NS
Pneumothorax	0	0	0	NS
Total complications	13 (9)	9 (8)	4 (13)	NS

PVC = peripheral venous line; CVC = central venous catheter; NS not satisfactory. ^a^ Values reported as number (%).

## Data Availability

The data presented in this study are available on request from the corresponding author.
